# Dissociable Modulation of Overt Visual Attention in Valence and Arousal Revealed by Topology of Scan Path

**DOI:** 10.1371/journal.pone.0018262

**Published:** 2011-04-06

**Authors:** Jianguang Ni, Huihui Jiang, Yixiang Jin, Nanhui Chen, Jianhong Wang, Zhengbo Wang, Yuejia Luo, Yuanye Ma, Xintian Hu

**Affiliations:** 1 State Key Laboratory of Brain and Cognitive Science, Beijing, People's Republic of China; 2 Kunming Institute of Zoology, Chinese Academy of Sciences, Kunming, Yunnan, People's Republic of China; 3 Institute of Biophysics, Chinese Academy of Sciences, Beijing, People's Republic of China; 4 Graduate School of Chinese Academy of Sciences, Beijing, People's Republic of China; 5 Key Laboratory of Mental Health, Institute of Psychology, Chinese Academy of Sciences, Beijing, People's Republic of China; 6 State Key Laboratory of Cognitive Neuroscience and Learning, Beijing Normal University, Beijing, People's Republic of China; Kyushu University, Japan

## Abstract

Emotional stimuli have evolutionary significance for the survival of organisms; therefore, they are attention-grabbing and are processed preferentially. The neural underpinnings of two principle emotional dimensions in affective space, valence (degree of pleasantness) and arousal (intensity of evoked emotion), have been shown to be dissociable in the olfactory, gustatory and memory systems. However, the separable roles of valence and arousal in scene perception are poorly understood. In this study, we asked how these two emotional dimensions modulate overt visual attention. Twenty-two healthy volunteers freely viewed images from the International Affective Picture System (IAPS) that were graded for affective levels of valence and arousal (high, medium, and low). Subjects' heads were immobilized and eye movements were recorded by camera to track overt shifts of visual attention. Algebraic graph-based approaches were introduced to model scan paths as weighted undirected path graphs, generating global topology metrics that characterize the algebraic connectivity of scan paths. Our data suggest that human subjects show different scanning patterns to stimuli with different affective ratings. Valence salient stimuli (with neutral arousal) elicited faster and larger shifts of attention, while arousal salient stimuli (with neutral valence) elicited local scanning, dense attention allocation and deep processing. Furthermore, our model revealed that the modulatory effect of valence was linearly related to the valence level, whereas the relation between the modulatory effect and the level of arousal was nonlinear. Hence, visual attention seems to be modulated by mechanisms that are separate for valence and arousal.

## Introduction

The emotional system is intrinsically significant to the survival of the organism. From an evolutionary perspective, emotional experience enables organisms to automatically evade threats in the environment, such as predators [1]. Emotionally salient stimuli inherently draw attention and receive preferential treatment in perceptual processing, particularly when attentional resources are limited and therefore have privileged access to awareness [2]. For instance, in a visual search task, fear-relevant pictures (e.g., snakes or spiders) are found more quickly than fear-irrelevant ones [3]. Nonetheless, the underlying mechanisms of this “pre-attentional” processing of emotional stimuli are poorly understood. However, emerging data have indicated that the emotion and cognition systems presumably closely interact in affecting behavior [4], [5], [6], [7].

Affective space is a theoretical system describing affective stimulus and experience and has been characterized by two principal dimensions, hedonic valence and arousal [8], [9], [10], [11]. Valence refers to the pleasantness/unpleasantness of an experience; arousal, on the other hand, is associated with the intensity of emotional activities and the calm/excited state of the body [10]. Though valence and arousal are two independent dimensions characterizing affective experiences [12], they are closely correlated [13], [14], i.e., both negatively and positively valenced stimuli would be rated as more arousing than neutrally valenced stimuli. However, little is known about the separable roles of valence and arousal in modulating cognitive operations like, for instance, overt visual attention, which is critical to understanding the mechanisms of interaction between emotion and cognition.

In this study, we investigated how these two distinct emotional dimensions modulate overt visual attention in scene perception. Mounting neurophysiologic evidence has revealed dissociated neural underpinnings of valence and arousal in human olfactory [12], gustatory [15] and memory systems [16]. These studies found that the amygdala is associated with the intensity of the stimulus, while the prefrontal cortex (PFC) and the orbitofrontal cortex are associated with valence. Because it is relatively difficult to dissociate these two dimensions in visual stimuli [12], [17], the related neural representations of valence and arousal in the visual system are still in dispute [18]. Recently, converging lines of brain imaging evidence have indicated the amygdala responds to arousal-salient visual stimuli, whereas the lateral and medial PFC are associated with valenced visual stimuli [19], [20], [21]. Employing the “Attentional Blink” paradigm, Anderson found that the arousal value, rather than the valence value, of emotional words is responsible for the preferential access of emotional stimuli to awareness [22]; this suggests that the arousal value plays a critical role in guiding visual attention. Maljkovic and Martini also reported that valence and arousal affect visual short-time memory independently in humans viewing a rapid stream of affective images [23]. Therefore, we hypothesized that valence and arousal play different roles in guiding visual attention as human subjects freely view affective images.

To test this hypothesis, we needed to overcome two technical difficulties. The first was dissociating these two tangled emotional dimensions in order to examine them separately. A related difficulty was minimizing the influence of other factors contributing to visual attention, from low-level image properties to top-down information (e.g., image familiarity). The second difficulty was characterizing the overt visual attention that is modulated by these two dimensions. In this study, we used complex color pictures selected from the International Affective Picture System (IAPS) as visual stimuli [14]. These pictures were categorized into two stimuli blocks, the Valence Block (VB) and the Arousal Block (AB), and a single emotional dimension dominated pictures in each block. In addition, pictures in each block were divided into three graded affective levels (High, Medium, and Low) to examine graded effects within each dimension. In the VB, for instance, pictures were categorized into three groups: High-Valenced (HV, positive), Medium-Valenced (MV, neutral) and Low-Valenced (LV, negative). All pictures in the VB had a medium arousal level, thereby decorrelating the two intimately interacting dimensions. To control for low-level image properties, we carefully distributed images in each block to balance spatial frequency [24], [25], luminance and complexity [26]. We also asked subjects to rate the familiarity of each image on a seven-point scale after viewing that image. Overt visual attention and saccadic eye movements are tightly coupled [27], [28], [29], especially in complex cognitive processes like reading, scene perception and visual search [27], [30]. Therefore, we used saccadic eye movements to characterize overt shifts of visual attention and scan path patterns to provide direct information about the modulation of visual attention.

Most of the existing research on the subject has employed discrete scan path metrics (fixation numbers, fixation duration, saccade amplitude, etc. [27], [31], [32]), to characterize scan path patterns. Quantitative models, like the string sequences model [33], [34] and stochastic process models [35], [36], have been used to describe scan paths. Although these approaches possibly reflect information about partial local patterns, global pattern information of the scan paths was neglected. These techniques are not sufficient to provide a comprehensive profile of scan path patterns, and, hence, they are insufficient to uncover the underlying cognitive significance. Here, we introduce a series of novel techniques to describe the global topological structure of scan paths. We modeled the scan-path as an un-directed weighted path graph. Because the path graph consists of vertices and the edges between them, it approximates the real scan path of eye movement trajectories. Global topology metrics of the scan-path reduced path graph (**SPPG**) were calculated. For instance, the Fiedler constant, which characterizes the algebraic connectivity of the graph, was calculated. We further employed spectral embedding approaches to transform scan paths into the Euclidean spaces 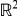 and 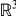, where these spectral embedded scan paths could be described in simple polynomial forms. Finally, we integrated the global topology information provided by the **SPPG** model with local pattern information from discrete local metrics to explore the separable roles of valence and arousal in modulating overt visual attention.

## Results

### Decorrelating Valence and Arousal

**Figure 1:**
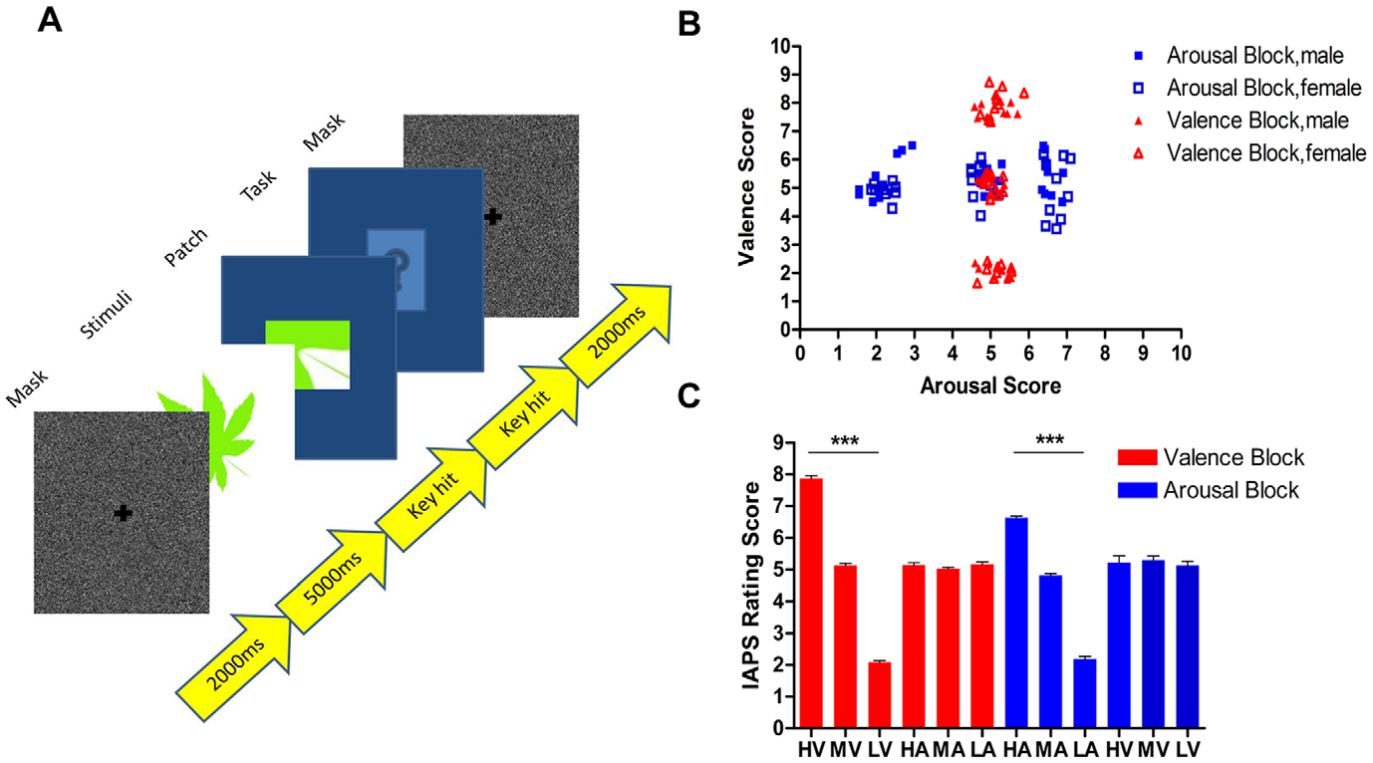
Experiment procedure and stimuli. (A) Flow chart of the experiment procedure. There were four consecutive stages in each trial: Mask (Gaussian-noise image), Stimuli (display of the target affective picture and monitoring of monocular eye position), Patch (patch image recognition task, to encourage free viewing) and Task (picture familiarity rating on a 7-point scale). (B) The scatterplot shows affective ratings of IAPS pictures in the valence block (VB, red triangles) and the arousal block (AB, blue squares) by females and males. Scores in the two dimensions were independent and decorrelated. (C) The bar graph shows valence ratings among three graded levels (HV, MV, LV) in VB (red bar), and arousal ratings among three graded levels (HA, MA, LA) in AB (blue bar). Three stars mark highly significant difference (p< 0.001, Kruskal-Wallis test).

Healthy subjects freely viewed 30 affective IAPS pictures in both a valence block (VB) and an arousal block (AB). Figure 1 shows the task procedure and the affective ratings of the pictures. Comparisons of rating scores (mean±std) in each block were performed by Kruskal-Wallis test. In the valence block, pleasant (7.87±0.40, HV), neutral (5.13±0.28, MV) and unpleasant pictures (2.08±0.22, LV) were rated significantly different in valence (p<0.0001), while arousal ratings in the valence block were at the calm level (5.11±0.29, p = 0.26) and not significantly different between valence levels. In the arousal block, arousal ratings of exciting (6.63±0.24, HA), calm (4.81±0.26, MA) and sleepy pictures (2.18±0.36, LA) were significantly different (p<0.01), while valence ratings in the arousal block were at the neutral level (5.21±0.69, p = 0.54) and not significantly different between arousal levels. Hence, the present study has dissociated the individual effects of valence and arousal. No low-level image properties such as spatial frequency, luminance or complexity showed significant differences within each dimension or between the two dimensions (both p<0.05). Picture familiarity ratings were also not significantly different between valence and arousal blocks, despite there being a marked difference within both the VB and AB (both p<0.01), due to the enhanced memory effect [8], [37]. Therefore, our stimuli dissociated the two emotional dimensions and excluded the possible contributions of low-level image properties and top-down information such as familiarity (Text S1).

### Path Graph Model of Scan Path

**Figure 2:**
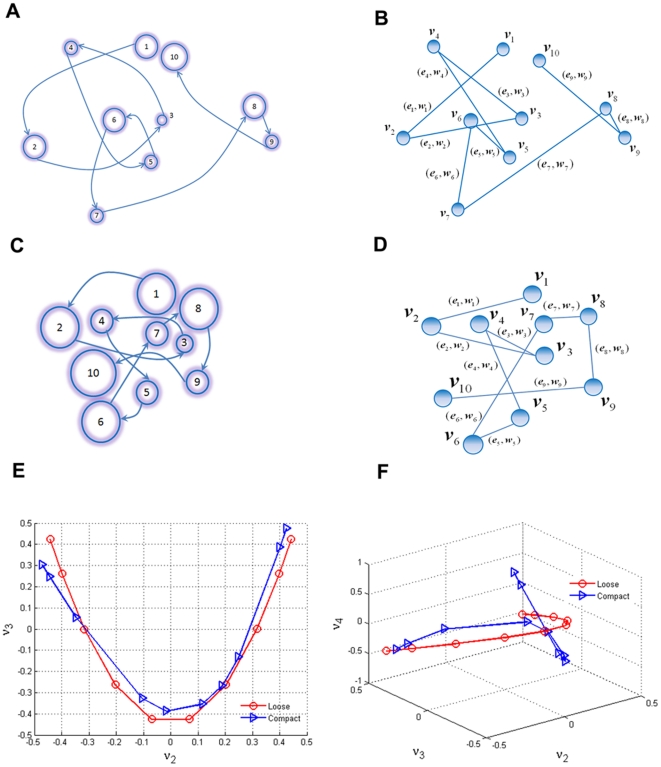
Path graph model of scan path. (A and C) Schemas that illustrate two scan paths with loose and compact patterns. The shaded circles with numbers denote fixation sequence. Larger circles indicate longer fixation durations. The arrow line between two fixations indicates a saccade. (B and D) Weighted undirected path graph models corresponding to real eye movement scan paths in (A) and (C), respectively. The vertices represent gaze fixations. Each edge is weighted by the Mahalanobis distance between two adjacent vertices. (E) Spectral embedding in 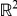 eigen-space. Two scan paths with different patterns were embedded into 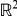 eigenspace by the orthonormal basis 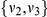 of its Laplacian matrix. (F) Spectral embedding in 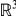 eigenspace. Scan-paths were embedded into 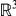 space by the orthonormal basis 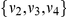 of its Laplacian.

We posit that a scan-path of eye movement in scene perception can be firstly approximated as a path graph. A path graph refers to a connected graph with only one edge between any pair of consecutive vertices [38]. Geometrically, the scan path is equivalent to the path graph if one takes fixation as the vertex and inter-fixation saccades as the edges, although real saccade trajectories deviate from straight vectors [39], [40], [41], [42]. To model a real scan-path, two hypotheses were proposed: firstly, the edge of path graph was weighted by eye movement parameters. Secondly, the scan path was modeled as the undirected path graph instead of the directed graph because of the computational advantages of this approach [38]. Thus, the scan-path reduced path graph (**SPPG**) was modeled as weighted un-directional path graph. See (A-D) in Figure 2.

In the **SPPG** model, path graph 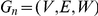, where 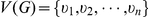 denotes the vertex set with *n* vertices, 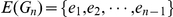 denotes the edge set with *n*-1 edges, and 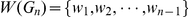 denotes the weight set with *n*-1 weights. Here, we introduce topological metrics characterizing the global features of the scan-path of eye movement.

#### Weight

The edge weight of the path graph is generally measured by correlation, mutual information or distance [43] between adjacent pairs of vertices. In this study, nine dynamic parameters of scan path were obtained by an EyeLink 2000 eye-tracking device. These parameters were X (horizontal coordinate of the current fixation), Y (vertical coordinate of the current fixation), PUL (pupil size at the current fixation), DRA (duration of the current fixation), SAP (inter-fixation distance or current saccade amplitude), SAG (current saccade angle of the current fixation), AGV (average velocity of the current saccade), SDR (current saccade duration) and PKV (peak velocity of the current saccade). Therefore, the vertex g could be written as multi-dimensional vector, giving 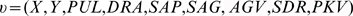. Weight could then be given by the function 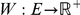, where 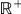 is the set of positive numbers. Hence, the weight could be given as



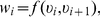
(1)where 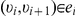, i = 1,2,⋯n-1.

In this study, the weight function employs the form of the Mahalanobis distance. This measurement eliminates the effects of distinct dimension units, and underscores the correlation of different dimensions in a multi-dimensional vector, which gives 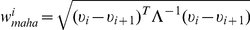, where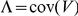 is the covariance matrix of vertices set 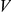. For statistics of the edge weights in VB and AB see Text S1.

#### Diameter

The scan path diameter, an invariant in graph topological structure, refers to the maximum shortest distance over any pair of vertices. A larger diameter in scan path indicates an elongated pattern. In the **SSPG** model, we defined the diameter 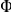 as the sum of weights, which gives



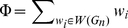
(2)


#### Laplacian Matrix

Given a weighted undirected path graph 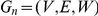, eigenvalues and eigenvectors of its Laplacian matrix 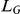 characterize the main topological structures of the graph. The Laplacian of 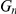 could be given as 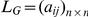, in which 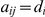, if 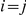, where 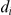 was the sum of degrees of the 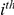 vertex; and 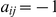, if 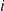 and 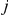 were adjacent; otherwise 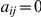. Regarding of the spectrum of the Laplacian matrix,



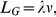
(3)


where 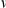 is the eigenvector of the corresponding eigenvalue 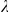 in 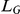. Specifically, since the Laplacian is a positive semi-definite matrix, the Perron-Frobenius theorem ensures all its eigenvalues are positive real numbers [44]. Hence, that gives,



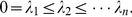
(4)


The second smallest eigenvalue 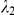, namely the Fiedler constant, refers to ‘algebraic connectivity’ of the graph [38], [44], [45], [46].

#### Fiedler

In the **SPPG** model, this topological metric characterized the global connectivity (compactness) of the path graph. A large Fiedler refers to a compact topological pattern, indicating dense attentional allocation in scene perception, while a small Fiedler refers to an elongated pattern, which indicates loose attentional allocation. If 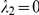, this indicates a disconnected graph.

#### Spectral Embedding

Scan paths consist of stochastically scattered fixations. To transform the scan path into a predictable pattern, we embedded it into a new m-dimensional Euclidean space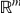. The embedding represents the graph in a target coordinate system with its vertices in a new layout while the graph topology remains the same. More specifically, vertices in the embedded graph will have the same adjacent relationships. Mathematically, the vertex 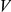 in graph 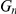 was mapped into 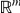 by the representation function 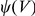, where 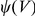, an m-dimensional row vector served as the new coordinate for 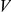 in the embedded space 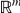. The representation was balanced if 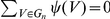. Because there was a central fixation bias or a ‘center of gravity’ in the scan path pattern [47], [48], our model assumed 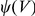
was balanced; therefore, no information was lost in the embedding [38]. Thus the path graph 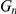, embedded into 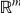 by 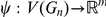 gives



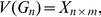
(5)


where 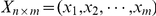 is the embedded matrix with m-dimensional coordinates of 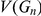 in 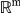. If the columns in 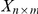 are not linearly independent, the embedding may not sufficiently determine all topological properties of the graph; therefore, we assumed 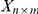 was linearly independent and orthogonal.

To assess the embedding stability, energy 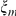 was defined as the sum of weighted distances in adjacent vertices over the embedded space 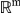, 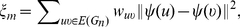
(6)


Where ∥∘∥ denotes the Euclidean distance and 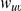 is the weight on edge 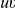 of two adjacent vertices u and 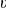. Lower energy indicated a more stable representation [38]. Theories of algebraic graphs state that the minimum energy of a balanced orthogonal representation equals thesum of the first m+1 eigenvalues (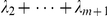) by the representation function consisting of the eigenvector of the Laplacian matrix [38]. Thus, the spectral embedding matrix of scan path 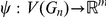 can be written as 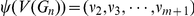. In fact, because these eigenvectors were mutually orthogonal vectors of magnitude equal to one, they formed the ortho-normal basis for the m-dimensional Euclidean space 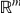. To ensure a unique embedding matrix, we assumed that all eigenvectors used in the embedding matrix should satisfy the constraint 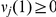, j = 1, 2, ⋯, m; otherwise we used (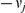) as a substitution.

Eigenvectors 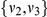, which correspond to eigenvalues 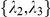, of the Laplacian, provided the space coordinate for embedding a scan path into a two-dimensional eigenspace 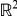. Figure 2E shows the 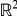 spectral embedding transformed scan paths as quadratic curves. Furthermore, the embedded curves could be fitted by the quadratic function 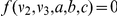 in the vertex form, which gives 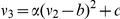. The fitted quadratic function indicated a simple but efficient way to describe the embedded path graph. Geometrically, the coefficient ***α*** denotes opening orientation (upwards or downwards) and opening width of the parabola. According to the constraint condition 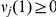, coefficient ***α*** should be a positive number in our model; hence all embedded scan paths open upwards. The coefficient ***b*** determines the symmetric axis position, and coefficient ***c*** denotes the vertical position of the fitted quadratic curve. Likewise, eigenvectors 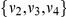 of the Laplacian formed an orthonormal basis, embedding the scan path into cubic curves (see Text S1) in 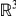 embedding. These cubic curves could be fitted as 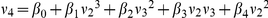.

### Shifts of Pupillary Reflex Curves by Valence and Arousal

**Figure 3:**
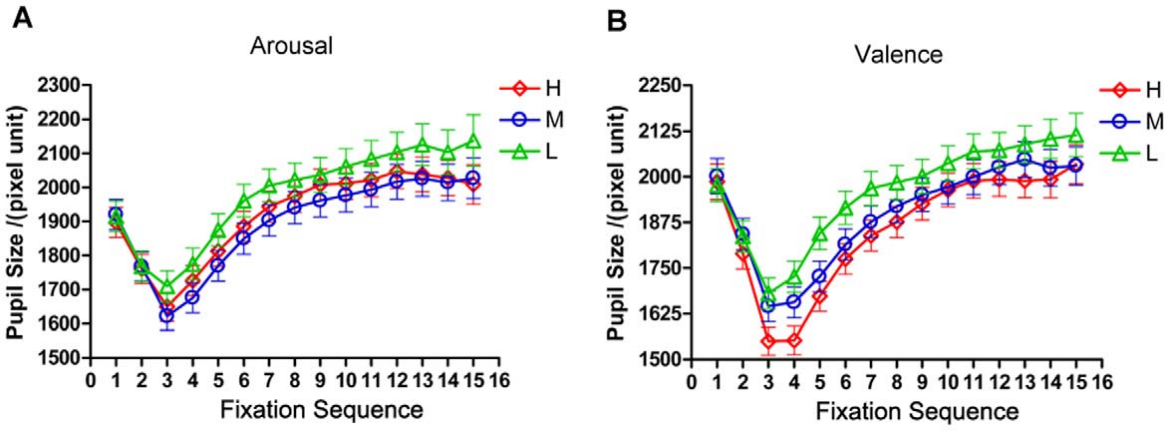
Shifts of pupillary reflex curves by valence and arousal. The pupillary reflex curve is plotted by the pupil diameter of the first 15 consecutive fixations (duration ≈5 seconds) in the scan path and pooled over 22 subjects. (A) Shifts of the pupillary reflex curves by emotional arousal (p<0.01, repeated ANOVA). Pupil diameter while viewing low (LA) and high arousal pictures (HA) is larger than viewing medium arousal pictures (ML), indicating a non-linear effect of arousal (F(1,14) = 66.022, p<0.01). (B) Shifts of the pupillary reflex curves by hedonic valence (p<0.01, repeated ANOVA). Pupil size while viewing valenced pictures is linearly related to the valence level, suggesting a linear effect of valence (F(1,14)  = 66.022, p<0.01).

Pupil size was monitored during viewing of affective pictures. The pupillary reflex curves showed pupil diameter constricted due to a reflex response to the initial light in the first three or four fixations (Figure 3) and then gradually dilated to normal size. The pupillary curve was shifted significantly compared to medium by high or low affective levels of valence and arousal (both p<0.01, repeated ANOVA), indicating the affective pictures elicited activation of the sympathetic nervous system [49]. In line with Bradley et al. [49], the pupil diameter was found to be a measure of emotional arousal, in that changes in size were smaller for neutral pictures. More importantly, we found a non-linear trend in the arousal effect (F(1,14) = 66.02, p<0.01), where low arousal pictures (AL) elicited larger pupil sizes compared to high arousal pictures (AH); and medium arousal pictures (AM) elicited the smallest pupil sizes. In addition, we found the effect of hedonic valence is linear, as pupil size is augmented as the hedonic valence level drops (F(1,14)  = 66.02, p<0.01). Therefore, our results suggest that the pupil diameter works as a measure of both hedonic valence and emotional arousal; in addition, the effects of hedonic valence and emotional arousal presumably operate on different principles (linear in valence vs. non-linear in arousal).

### Dissociation in Topological Metrics

**Figure 4:**
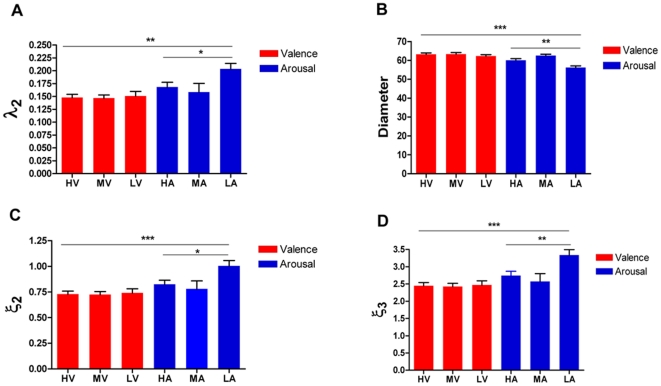
Topological metrics of scan paths. (A) Fiedler constant for the three affective levels (H, M, L) of the valence and arousal blocks. The scan paths of arousal pictures have larger Fiedlers compared to scan paths of valenced pictures. Within the arousal block, the Fiedler is significantly different across three graded levels, indicating a non-linear effect of arousal. (B) Diameter of path graph. The average diameter of path graph for valenced pictures is larger than that for arousal pictures. Significant within-block differences could be observed in arousal block, indicating non-linear effect of arousal. (C and D) Energy metrics of 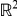 and 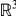
embeding. 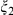 and 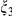 showed differences between the valence and arousal block. For arousal pictures, there is a significant within-block difference, suggesting a non-linear arousal effect. Star marks significant difference (one star: p<0.05; two stars: p<0.01 and three stars: p<0.001; by ANOVA test).

We excluded scan paths with less than five fixations from the SPPG model, because subjects may not have been viewing the picture, or the eye tracking device may have lost the pupil. The mean scan path diameter in valenced pictures was remarkably larger (p<0.01, Welch's t-test) than that in the arousal pictures (Figure 4B). The diameters did not show significant differences between the three valence levels (p = 0.57, ANOVA). In contrast, the diameters showed marked differences between the three arousal levels (p<0.01, ANOVA), where low arousal pictures had shorter diameters compared to medium and high arousal pictures (p<0.01 and p<0.001, respectively, Turkey's multiple comparison test). Furthermore, trend analysis revealed a significant quadratic trend (F(1, 214) = 29.06, p<0.01) in arousal levels, indicating a non-linear effect of the arousal dimension.

In the valence block, the Fiedler (±s.e.m.) was not significantly different between the three levels (p = 0.92, ANOVA). Interestingly, within the arousal block, the Fiedler showed a significant difference (p<0.05, ANOVA). Tukey's test revealed that the Fiedler for medium arousal pictures (0.1586±0.0170, N = 218) was markedly smaller than that for low arousal pictures (0.2036±0.0109, N = 218, p<0.05), whereas the Fiedler for high arousal pictures (0.1684±0.0091, N = 217) showed no significant differences compared to that for medium and low arousal pictures. Trend analysis revealed a significant non-linear trend over the three arousal levels (F(1,216) = 9.73, p<0.01). In addition, we found the Fiedler for valenced pictures was remarkably smaller than for arousal pictures (p<0.01, Welch's t-test). This result indicates valence salient pictures elicited loose scanning patterns compared to arousal salient pictures (Figure 4A and Text S1).

In the valence block, the representation energy metrics 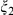 and 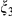 were pronouncedly lower than those in the arousal block (both p<0.01, by Welch's t-test, Figure 4C–D). In the valence block, the two energy metrics showed no significant difference between the three valence levels. In contrast, they both showed marked differences between affective levels in the arousal block (p<0.05 and p<0.01 respectively, ANOVA). Within the three graded arousal levels, 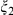 had no significant quadratic trend (F(1, 216) = 2.56, p = 0.11); however, 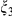 exhibited a marginally significant quadratic trend (F(1, 216) = 3.69, p = 0.056). These results suggest that embedded scan paths in the valence block were more stable than in the arousal block and indicated a possible non-linear arousal effect.

**Figure 5:**
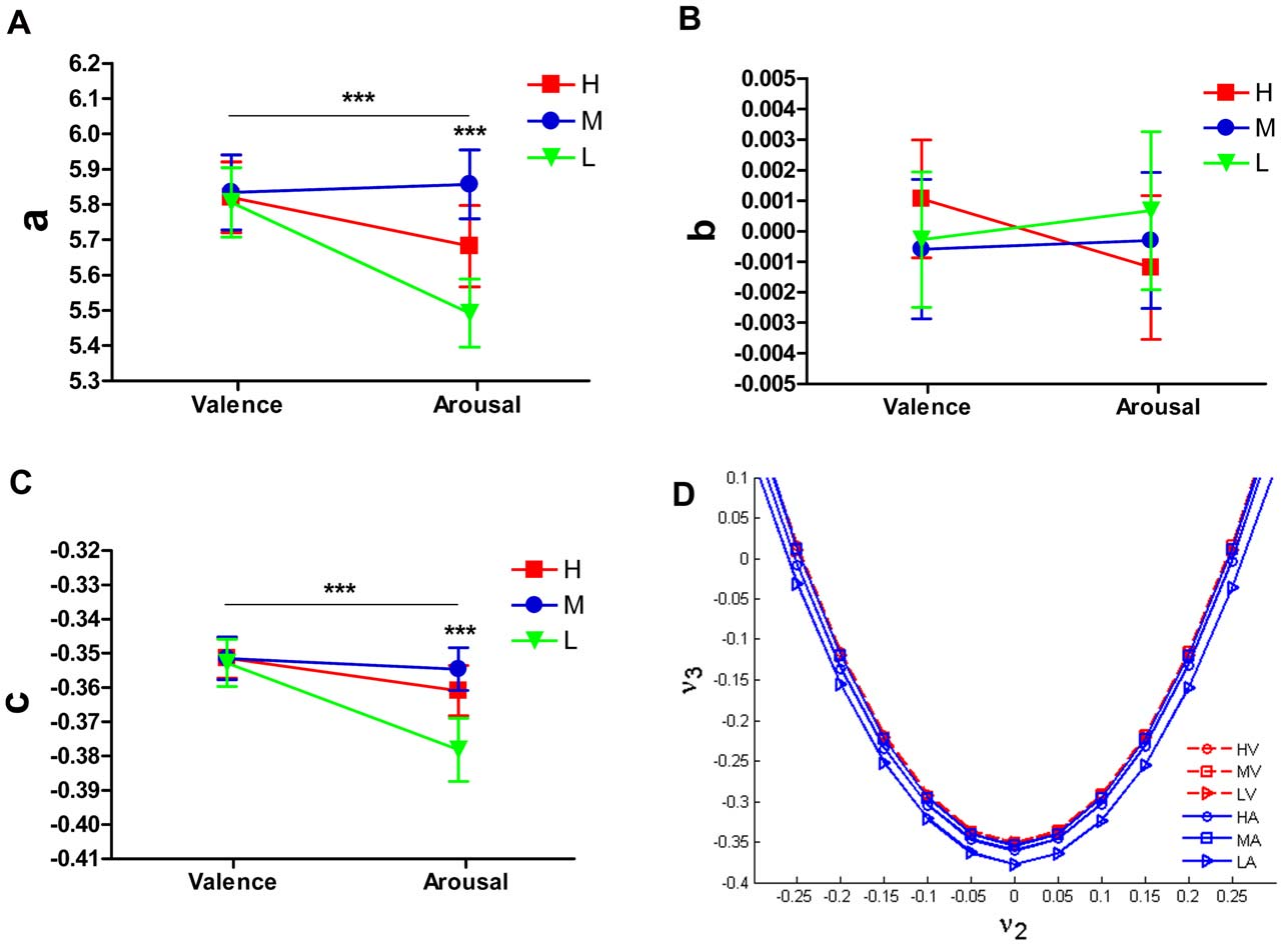
Quadratic curves of 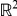 spectral embedding. The embedded path graph was fitted by the quadratic function in the vertex form of 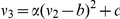. (A) Coefficient ***a*** is positive, therefore all embedded quadratic curves opened upwards. Fitted quadratic curves in embedded path graph of valenced pictures have larger open width; the coefficient ***a*** is significantly different across three arousal levels, indicating a non-linear effect of arousal. (B) Coefficient ***b*** represents the symmetry axis of the fitted parabola. There was no significant difference between the valence and arousal blocks for coefficient ***b***. (C) Coefficient ***c*** represents the height (vertical position) of the fitted quadratic curves. The fitted curves have larger coefficients ***c*** in valenced pictures and were significantly different within graded arousal levels, indicating a non-linear arousal effect. (D) Embedded quadratic curves in each affective level of two blocks. Stars mark significant difference (two stars: p<0.01 and three stars: p<0.001; by ANOVA test). Here 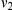 and 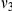 denotes the 2^nd^ and 3^rd^ eigenvector of the Laplacian matrix, respectively.

**Figure 6:**
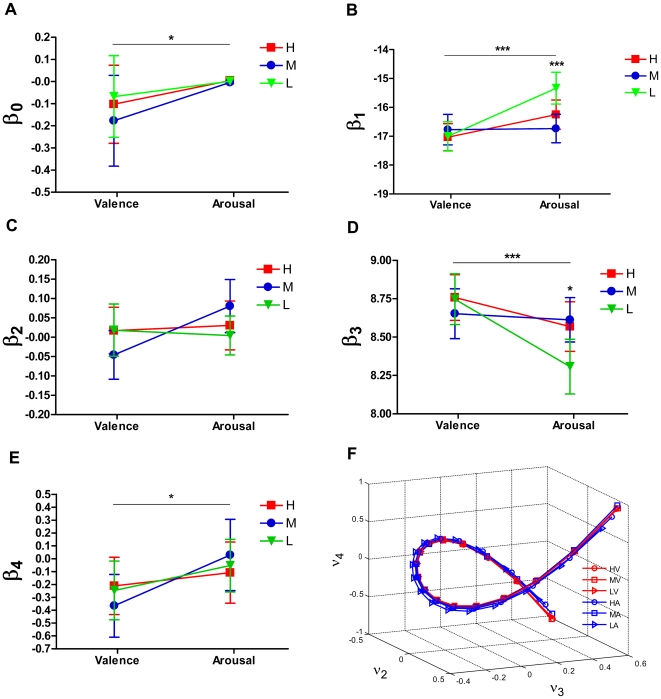
Cubic curves of 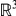 spectral embedding. 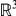
 The embedded path graph was fitted by the cubic function in the form of 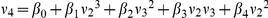. (A - E) Coefficients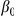,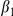,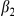,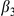 and 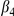 were compared between the two blocks and within each block. There are significant differences between valenced pictures and arousal pictures for the coefficients 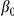,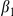,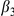 and 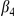, where coefficients 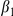 and 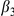 were different in graded arousal levels, indicating a non-linear effect of arousal. (F) The embedded curves in each affective level of valence and arousal. Star marks significant differences (one star: p<0.01; two stars: p<0.01 and three stars: p<0.001; by ANOVA test). Here 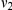, 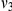 and 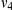 denote the 2^nd^, 3^rd^ and 4^th^ eigenvector, respectively.

Spectral embedding further revealed the dissociation of valence and arousal. Figure 5 shows the coefficients of the fitted curves in the 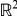 spectral embedding. The coefficient ***a*** in the VB was significantly larger (p<0.01, Welch's t-test) than in the AB, indicating a larger opening width of the parabola in valenced images. Within the AB, the coefficient ***a*** showed a marked quadratic trend over the three arousal levels (F(1, 216) = 23.14, p<0.01). The coefficient ***c*** was larger in the VB than in the AB (p<0.01, by Welch's t-test). The coefficient ***c*** also showed a significant quadratic trend over the three levels of arousal (F(1, 216) = 13.35, p<0.01). However, the coefficient ***b*** showed no significant difference between the VB and AB (p = 0.71, Welch's test), and within each dimension (p = 0.52 in VB and p = 0.56 in AB, ANOVA). Figure 6 illustrates the coefficients of the fitted cubic curves in the 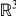 spectral embedding. The coefficients 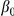,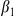,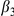 and 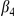 were significantly different between the VB and AB (p<0.05, p<0.01, p<0.01 and p<0.05, respectively, Welch's t-test). The within-block difference in the arousal block was significant for coefficients 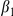 and 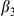
(p<0.01 and p = 0.02 respectively, ANOVA) and showed a quadratic trend over the three arousal levels (F(1, 213) = 10.95, p<0.01 and F(1, 213) = 3.92, p = 0.049, respectively), indicating a non-linear effect of arousal. Coefficient 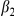 showed no significant difference between the two affective picture blocks (p = 0.11, Welch's test).

### Kinetic Scan Path Metrics

We found valenced affective pictures (VB) elicited faster, global scanning, while arousal affective pictures (AB) elicited slower, local scanning (Text S1). The average saccade velocity (AGV, mean±s.e.m.) was approximately 127.46±0.81(deg/sec) in the VB, and this was significantly greater than that in the AB (120.22±0.73, deg/sec, p<0.01). The fixation duration (DRA) in the VB (229.50±1.22, ms) was significantly smaller (p<0.01) compared to that in the AB (245.10±1.45, ms). The number of fixations in the scan path (FNum) for arousal pictures (16.04±0.13) was less than that for valenced pictures (16.75±0.14, p<0.01). The saccade amplitude (SAP) in VB (6.75±0.06, deg) was markedly larger than that in AB (6.13±0.05 deg, p<0.01). The peak saccade velocity (PKV) in VB (376.81±3.35, deg/sec) was also significantly larger than that in AB (363.69±3.25, deg/sec, p = 0.005). PUL (pupil size), SAG (saccade angle) and SDR (saccade duration) showed no significant differences between valence and arousal. These comparisons were performed by Welch's t-test.

We performed trend analyses to examine the effects of arousal and valence on kinetic metrics. DRA, FNum, PKV and SDR showed significant differences within the arousal block (all p<0.01, ANOVA) and exhibited significant quadratic trends (DRA: F(1,3308) = 33.78, p<0.01; FNum: F(1,219) = 32.01, p<0.01; PKV: F(1,3089) = 8.91, p = 0.003; SDR: F(1,3089) = 18.91, p<0.01), indicating a non-linear effect in the arousal dimension. On the other hand, SAP, PKV and SAG showed remarkable differences within the valence block (p = 0.04, p = 0.03,and p<0.01, respectively, ANOVA). The peak velocity and saccade angle exhibited significant linear trends (PKV: F(1,3450) = 6.24, p = 0.01; SAG: F(1,3450) = 13.62, p<0.01), indicating a linear effect in the valence dimension, whereas the saccade amplitude showed significant quadratic trend (SAG: F(1,3450) = 5.14, p = 0.02). We have observed the shifts of pupillary reflex curves elicited by valence and arousal, and here we found the pupil size (PUL) showed significant difference within both arousal pictures (p<0.01, ANOVA) and valenced pictures (p = 0.0001, ANOVA). In the valence dimension, Turkey's test revealed a significant linear trend over HV, MV and LV (F(1,3667) = 88.19, p<0.01). In the arousal dimension, pupil size was larger at the high level compared to the medium level (p<0.01), but smaller compared to the low level (p<0.01), exhibiting a significant quadratic trend over HA, MA, and LA (F(1,3308) = 6.57, p = 0.01).

### Multivariate Linear Model

We build stepwise multivariate linear regression models for the Fiedler and spectral embedding metrics. There were eight independent scan path metrics: fixation number (FNum), diameter (Φ), skewness of edge weight distribution (SKW), kurtosis of edge weight distribution (KUR), average saccade velocity (AGV), saccade amplitude (SAP), saccade angle (SAG) and peak velocity of saccade (PKV). Table 1 describes the results of the model (see Text S1 for the partial regression plot). As can be seen in Table 1, scan paths of more fixations in VB were related to smaller Fielders, indicating a loose pattern. In contrast, scan paths of positively skewed and leptokurtic weight distributions were related to larger Fiedlers and, hence, predicted a compact pattern. Coefficient ***a*** was predicted by FNum and SAP (R^2^ = 0.89, p<0.01, F-test) in VB and by FNum and SKW (R^2^ = 0.79, p<0.001, F-test) in AB. Coefficient ***b*** was regressed significantly by FNum, SKW, SAP and PKV (R^2^ = 0.31, p<0.01, F-test) in AB. In predicting the coefficient ***c***, FNum and AGV explained most of the variability (90.20%, p<0.01, F-test) in VB; while the diameter and the KUR contributed 82.70% of variability (p<0.01, F-test) in AB. In terms of the 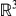 embedding curve metrics (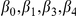), FNum, diameter and the SKW explained most of the variability. In summary, the multivariate linear model provided a possible relationship between the global topological metrics and kinetic scan paths metrics.

**Table 1:** Results of the multivariate linear model.

Dependent	Block	R^2^	Independents and partial coefficients						
			FNum	Φ	SKW	KUR	AGV	SAP	PKV
Fielder	VB	0.864^1^	−0.045^1^	0.007^2^			−0.001^2^		
	AB	0.703^1^		−0.009^1^	0.001^1^	0.026^2^			
*a*	VB	0.894^1^	0.166^1^					−0.037^3^	
	AB	0.790^1^	0.208^1^		−0.288^3^				
*b*	AB	0.309^1^	−0.002^1^		0.035^3^			−0.002^2^	2e-5^3^
*c*	VB	0.902^1^	0.010^1^				0.0001^2^		
	AB	0.827^1^		0.003^1^		−0.006^1^			
*β*_0_	AB	0.822^1^	0.240^1^		0.354^3^				
*β*_1_	VB	0.798^1^		−0.246^1^					
	AB	0.889^1^	−0.877^1^		−1.090^3^				
*β*_3_	VB	0.690^1^		0.067^1^					
	AB	0.822^1^	0.240^1^		0.354^3^				
*β*_4_	AB	0.111^1^			0.598^1^				

The Fiedler and spectral embedded metrics of scan path were predicted by main scan path parameters in a stepwise multivariate linear regression model. VB, the valence block; AB, the arousal block; **R^2^**, the adjusted coefficient of determination; FNum, the fixation number; Φ, the scan path diameter; AGV, the average velocity of saccade; SKW, the weight skewness; KUR, the weight kurtosis; SAP, saccade amplitude; PKV, the peak velocity of saccade.

1 p<0.001, by F-test for the significance of the regression model or by t-test for the significance of partial coefficients.

2 p<0.01, by t-test for the significance of partial coefficients.

3 p<0.05, by t-test for the significance of partial coefficients.

## Discussion

This research investigated the roles of valence and arousal in modulating overt visual attention in scene perception. We dissociated two emotional dimensions in two stimulus blocks, thereby separating the effects of valence and arousal. We also carefully balanced low-level image properties such as spatial frequency, luminance and complexity. These low-level image properties showed no significant differences within or between the stimuli blocks of valence and arousal thereby excluding their possible contribution to the dissociation effect. We built a scan path reduced path graph (SPPG) model to characterize the global topological structure of scan path.

Two main results were obtained: Firstly, scan paths exhibited substantially distinct patterns between the hedonic valence pictures and the emotional arousal pictures. The Fiedler revealed a more compact scan path pattern in arousal pictures compared to valenced pictures; hence, our results imply separate mechanisms by which these two emotional dimensions modulate visual attention. Secondly, the affective effect of the valence dimension was linear, whereas the effect of the arousal dimension was nonlinear. These findings show that different mechanisms of information processing underlie the modulation of visual attention by valence and arousal and provide a new perspective on the mechanisms behind emotion-cognition interaction.

### Dissociation of Valence and Arousal

It has been proposed that the valence dimension has an underlying motivational basis [50]. Two motivational brain systems account for hedonic valence: the appetitive system facilitates approach-related behavior toward pleasant experiences, while the aversive system facilitates withdrawal-related behaviors away from unpleasant experiences [9], [51]. Emotional arousal, or the intensity of emotion, reflects the activation of the neural system. Arousal is usually measured by increases of blood flow, de-synchronization of electroencephalographs, the amplitude and latency of event related potentials, or pupil diameter, skin resistance and heart rate [49], [52]. Emerging evidence from brain imaging [12], [15], [16], [17], [53], [54], [55], [56] and ERP studies [57], [58], [59], [60], [61] has shown the neural underpinnings of valence and arousal are disassociated in the human olfactory, gustatory, and memory systems. In line with these neurophysiologic studies, our data revealed separate mechanisms by which valence and arousal modulate overt visual attention.

We examined the global metrics of scan paths and found more compact scan path patterns in arousal pictures than in valence pictures. The algebraic connectivity of the path graph (the Fiedler) was significantly larger for the arousal pictures than for the valence pictures. Furthermore, the diameters of the scan paths for the valence specific stimuli were remarkably smaller than those for the arousal-specific stimuli. The spectral embedding of scan paths also revealed differences in hedonic valenced pictures and arousal pictures. We also examined this dissociation effect with scan path kinetic metrics. The number of fixations, average saccade velocity, saccade amplitude and peak saccade velocity were greater in the valence block than in the arousal block (Text S1), while the fixation durations were shorter for valence specific picture than arousal specific pictures. Therefore, our results showed faster and larger shifts of attention in valence specific images than in arousal specific images.

From the discussion above, we may draw the conclusion that the affective arousal dimension elicits more local scan patterns, denser attention allocation and deeper information processing in scene perception compared to the valence dimension. In fact, emerging studies have pointed out that arousal, rather than valence, may play a pivotal role in guiding attention [55], [59], [62], [63]. In line with our study, Anderson reported that it is the arousal dimension, not the valence dimension, that is responsible for attentional blink sparing, indicating arousal decreases the attentional threshold for consciousness [22]. Schupp et al. have demonstrated the emotional intensity of stimuli, rather than their pleasantness, dominates the selective processing of affective contents when pictures are presented briefly [59]. From an evolutionary perspective, arousal is intimately associated with the survival of organism; hence it presumably plays a leading role in guiding attention.

### Linear versus Nonlinear Effect

**Figure 7:**
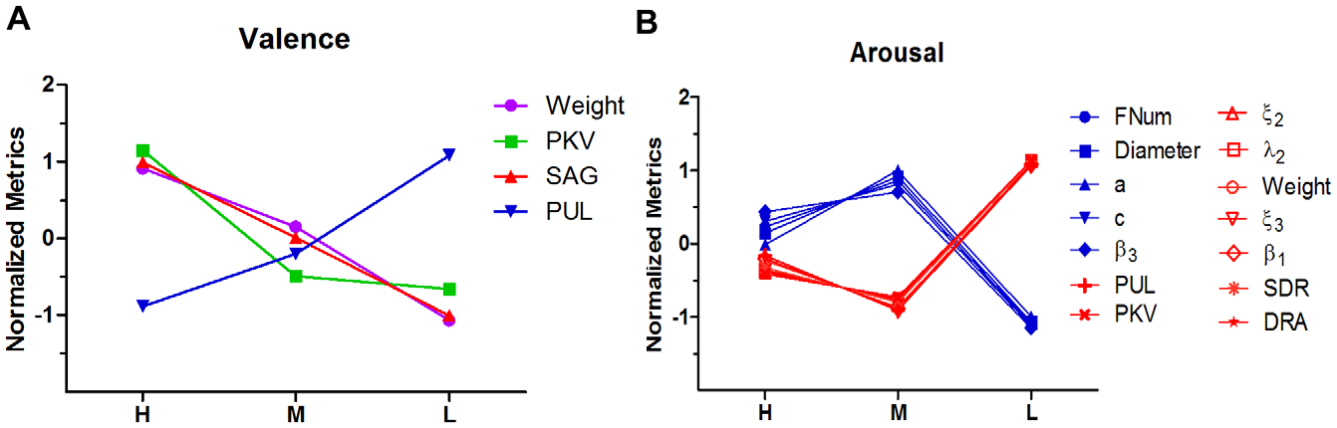
Linear valence effect and non-linear arousal effect. (A) Scan path found a significant linear trend across three graded valence levels (HV, MV and LV) in these metrics: edge weight (p = 0.02), peak velocity (PKV, p = 0.01), saccade angle (SAG, p<0.01) and pupil size (PUL, p<0.01), indicating a linear hedonic valence effect. (B) Scan path found a quadratic trend across three graded arousal levels (HA, MA and LA) in these metrics: fixation number (FNum, p<0.01), diameter of scan path (Diameter, p<0.01), coefficient ***a*** (p<0.01), coefficient ***c*** (p<0.01), PUL (p = 0.01), PKV(p<0.01), 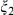
(p = 0.11), 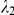
(p = 0.13), weight(p = 0.07), 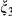
(p = 0.056), 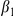
(p<0.01), saccade duration (SDR, p<0.01) and fixation duration (DRA, p<0.01). By F-test.

The dissociated neural representations of valence and arousal suggest that their information processing mechanisms operate in different ways. This study investigated the graded affective effect in each dimension and showed that the modulatory effect of valence was linear, whereas the modulatory effect of arousal was nonlinear (Figure 7).

In the valence block, we observed that as the valence level increased linearly from the low to the high level, metrics such as the edge weight, peak velocity, time course curves of peak velocity (TCC_PKV) and saccade angle showed positive linear trends, while the pupil size and papillary reflex curves showed negative linear trends. Our data therefore suggest that the overarching modulatory effect of the valence dimension works linearly (Figure 7, Text S1 and Table S1).

On the other hand, the modulatory effect of the arousal dimension displayed nonlinear trends as the arousal level increased from LA to HA (Figure 7, Text S1 and Table S2). Most of the scan path metrics exhibited quadratic trends over the three graded arousal levels. More specifically, there were two categories of quadratic trends. In the first category the effect at the medium level was greater than that at the high level, while the effect at the high level was greater than that at the low level (MA > HA > LA). Metrics such as the diameter, fixation number, coefficient ***a***, coefficient ***c***, and coefficient 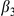 fit this category. The second category displayed the reverse trend (MA < HA < LA) and included such metrics as the edge weight, 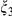, 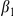, saccade duration, fixation duration, pupil size, peak velocity, pupillary reflex curve, fixation duration, peak velocity and saccade duration.

The neural underpinnings of arousal presumably involve the amygdala [19] and dorsomedial PFC [64]. Sabatinelli and colleagues have reported a positive linear relationship between the arousal quality of visual stimuli and activation of the amygdala [65]. The nonlinear trends shown for the arousal effect suggested that high arousal level (alert body state) and low arousal level (less activated body state), presumably have similar influences compared to the medium arousal level (calm body state). However, the underlying mechanism of this non-linear effect remains unclear. One possible explanation is that both the high and low arousal levels indicate a change in body state (alarmed and less-activated) compared to the medium level (calm state). This suggests that the overarching modulatory effect of the arousal dimension is not associated with the absolute arousal level, but related to the difference relative to the medium arousal level.

### Model Evaluation

The familiarity rating scores of pictures showed significant differences over graded affective levels of each dimension in this study. Evidence from visual search [66], [67] and recollection [68] shows effective processing of familiar stimuli. This raises the possibility familiarity could contribute to the observed dissociation between valence and arousal. We discount this possibility for several reasons. Firstly, there was no significant difference in familiarity scores between the valence and arousal blocks (Text S1). Secondly, topological metrics showed no within block difference in valence, while the picture familiarity score did differ across the three affective levels of valence. Thirdly, the familiarity effect may simply be an epiphenomenon of the emotional memory effect [37], [69], [70]. Overall, the familiarity factor probably did not contribute to the dissociation between the two emotional dimensions. One possible drawback of this study was that we did not measure skin conductance to index sympathetic or parasympathetic nervous system activation during viewing of the affective pictures [49]. This would have provided supporting evidence of induced emotional experiences. Yet the pupillary reflex curves were sufficient to document the emotional effects induced by the stimuli. Lastly, since we did not directly measure the layout of key objects in each image, we could not exclude the possibility that the position of emotion-eliciting objects affected scan paths patterns. However, the low-level image property statistics of spatial frequency, luminance or complexity in this study were not significantly different, which provides indirect evidence that the influences of key object layout were very limited.

Approaches involving the measurement of eye movements provide a promising method for probing the brain function. As a novel and powerful technique for modeling eye movement scan path, the algebraic graph theory-based path graph model of scan path has several noticeable merits: (a) The **SPPG** model incorporated almost all the important dynamic information from the saccadic eye movement. Thus, this model could sufficiently approximate real scan paths without loss of critical information. (b) The traditional method of describing scan paths by isolated metrics splits internal associations of underlying pattern information. Topological metrics produced by the **SPPG** model provide global measures for scan paths, and thus attenuate variability in the stimuli. (c) The topological metrics and spectral embedding of scan paths reduces complicated eye movement scan paths into comparable curves with objective and robust metrics. Improvements of the current **SPPG** model could be made by extending spectral embedding to high dimensional space 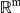 (m>3). Overall, this study combined both the local information and the global information from scan paths, provided a powerful tool for eye movement and cognition research.

## Methods

### Ethics Statement

The protocol of this study was approved by the Ethics Committee in Institute of Cognitive Neuroscience and Learning, Beijing Normal University. Subjects were informed with written consent. This study was compatible with Code of Ethical Principles for Medical Research Involving Human Subjects of the World Medical Association (Declaration of Helsinki).

### Participants

Twenty-two healthy volunteers (postgraduate students) were recruited (12 female and 10 male, 20–30 years old) from the Kunming Institute of Zoology, CAS. Subjects had normal or corrected-to-normal vision. All subjects were paid Y20. All Subjects were initially naive to experiment, but received training to familiarize them with the task procedure before beginning the experimental task. No subjects had any history of mental disorder.

### Apparatus

An EyeLink 2000 Desktop eye tracking system (SR Research Ltd., Ontario, Canada) was used to present stimuli and record eye movements. Monocular eye position data was sampled at 2000 Hz. Stimuli were displayed on a 19 inch LCD monitor (DELL E198FPf, 37.5×30.5 cm, resolution of 1024×768 pixels, refresh rate of 60 Hz). The eye-to-screen distance and the eye-to-camera distance were 70 cm and 50 cm, respectively. Thus, to the subjects, the screen occupied approximately 30°×25° of visual angle, horizontally and vertically, respectively. Subjects' heads were immobilized by a chin-rest. Saccades were detected by three thresholds: a velocity threshold of 30 ^o^/s, an initial acceleration threshold of 8000^ o^/s^2^ and a displacement threshold of 0.15°. Fixation was defined as the time between two saccades.

### Stimuli

#### Valence and Arousal Ratings

Because gender differences were observed in responses to visual emotional stimuli [71], [72], two equivalent affective picture packages for male and female subjects were selected from the International Affective Picture System (IAPS) [26]. Valence was rated on a scale from 1 (most unpleasant) to 9 (most pleasant); arousal was rated in scale from 1 (sleepy, not at all arousing) to 9 (most exciting). Each subject had to finish two blocks (60 pictures): the valences block (AB, 30 pictures) with 10 pleasant (HV), 10 neutral (MV) and 10 unpleasant (LV) pictures categorized by valence score; and the arousal block (30 pictures): 10 exciting (HA), 10 calm (MA) and 10 sleepy (LA) pictures categorized by arousal score. Pictures were complex, color scenes involving animals, people, blood, mutilation, nature scenes, etc. Arousal ratings for valence block pictures were at the medium level (M = 5.1, SD = 0.29), and valence ratings for arousal block pictures were at the neutral level (M = 5.21, SD = 0.69). The Pictures' IAPS series numbers are listed in Table S3.

#### Low-level Image Properties & Familiarity

The feature-based factors of picture complexity and luminance showed no significant differences in the valence block (p = 0.24 and p = 0.18, respectively) or the arousal block (p = 0.67 and p = 0.81, respectively), Kruskal-Wallis test. Picture complexity was measured as the compressed image file size in kB [26]. Larger file sizes indicated more complex images. Picture luminance was calculated with Adobe Photoshop CS2 (Adobe Systems Inc., USA) in 0–255 gray scale. The spatial frequencies of images in the two blocks showed no marked differences (Text S1). Familiarity was rated on a 7-point scale (1 = least familiar, 7 = most familiar) by subjects during the task. Differences in the familiarity ratings were significant within the valence block (p<0.01) and as well as within the arousal block (p<0.01), but were not significant between the two blocks (Kruskal-Wallis tests).

### Task Procedure

Participants sat in a quiet dark house, with their head placed on chin-rest in front of the stimulus presentation screen. Before picture display began, the ‘9-point calibration’ program of the eye-tracking system was run to ensure that the EyeLink camera could capture the subject's pupil. Each subject had to finish two blocks (60 trials), beginning with the valence block (VB, 30 trails) and followed by the arousal block (AB, 30 trails). Subjects were allowed a rest interval between blocks, the duration of which was left to their discretion. At the onset of a trail, a Gaussian-noise image was displayed on screen for two seconds, during this period the subject was asked to fixate on a black center cross. Then a target affective picture (randomly presented without repetition) was displayed for five seconds. Subjects were asked to view the picture freely and the left eye was monitored during this period. Next, a patch image (any part of the affective picture, 250×200 pixels, selected randomly from 30 patches) was presented in the screen center [73]. Subjects were asked to press the key ‘0’ if they thought this patch was part of the target picture, or press the key ‘1’ otherwise. Subjects were instructed to respond as quickly as possible. Audio feedback was given (a ‘doo’ sound for incorrect responses and a ‘dee’ sound for correct responses). This patch-task encouraged subjects to freely view the picture because the patch image was randomly clipped out of the target picture and it was too small to be easily recognized if subject had just fixated at one place. The familiarity rating task followed this; subjects were asked to hit a number key as soon as possible to rate the target picture on a 7 point scale of familiarity (1 =  totally unacquainted, 7 =  completely familiar). A ‘9-point calibration’ program was performed every ten trials in each block. The duration of the entire experiment was approximately 40 minutes.

### Data Analysis

Path graph modeling and computation of topological metrics were performed on the MATLAB (Mathworks Inc., MA, USA) software platform. Curve fitting of spectral embedding path graphs employed the Curve Fitting Toolbox in MATLAB. Scan paths with fewer than five fixations could not be fitted by our model, hence these data were discarded. Statistical comparisons employed Welch's t-test, ANOVA and Tukey's post hoc test. Rating scores were analyzed with nonparametric Kruskal-Wallis tests. Trend analysis on graded affective effects was performed by F-test.

## Supporting Information

Text S1 **Supplementary figures.**
(PDF)

Table S1 **The linear effect in valence.** The linear valence effect was test by ANOVA and Turkey's post hoc test. H =  high affective level; M =  medium affective level; L =  low affective level. TCC_PUL  =  pupillary reflex curves; TCC_PKV  =  time course curve of PKV.
(PDF)

Table S2 **The nonlinear effect in arousal.** The nonlinear effect was test by ANOVA and Turkey's post hoc test. H =  high affective level; M =  medium affective level; L =  low affective level. TCC_PUL  =  pupillary reflex curve; TCC_DRA  =  time course curve of DRA; TCC_PKV =  time course curve of PKV; TCC_SDR  =  time course curve of SDR.
(PDF)

Table S3 **IAPS Series Number of Pictures.**
(PDF)
