# Supplementary material for: Dissociable Modulation of Overt Visual Attention in Valence and Arousal Revealed by Topology of Scan Path
Source: PLoS One. 2011 Apr 6;6(4):e18262. doi: 10.1371/journal.pone.0018262 (PMC3071806; doi:10.1371/journal.pone.0018262)
Supplement: Text S1 — Supplementary figures. (PDF) [file pone.0018262.s001.pdf]

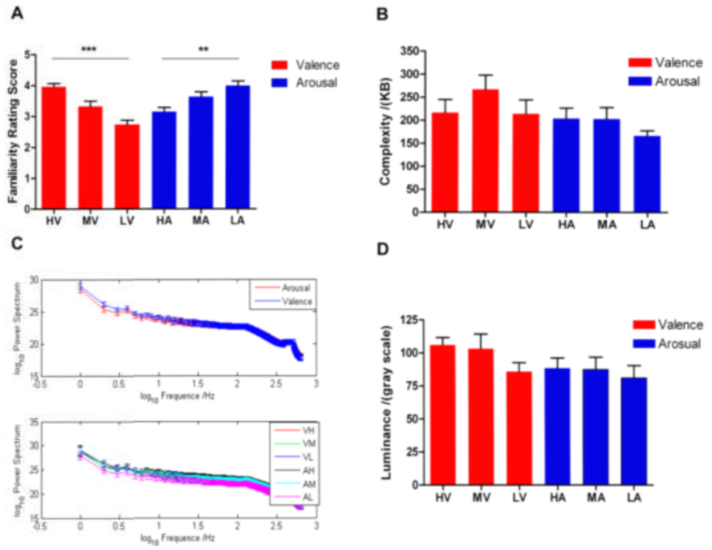

Figure S1. Familiarity ratings and low-level image properties. (A) Image familiarity ratings showed significant difference in high, medium and low level both within valence (red bar) and arousal block (blue bar). (B) Picture complexity was measured in Byte of the compressed image file. No significant difference of picture complexity is showed within the valence and arousal block. (C) The logarithmic frequency – logarithmic power spectrum plot in valence and arousal and their six affective levels. Bonferroni t-test showed no significant differences in power spectrum ($p > 0.05$). (D) Picture luminance was measured in 0-255 gray scale. Picture luminance showed no significant difference within each block. Star maker significant difference (one stars: $p < 0.05$; two stars: $p< 0.01$; three stars: $p< 0.001$; Kruskal-Wallis tests).

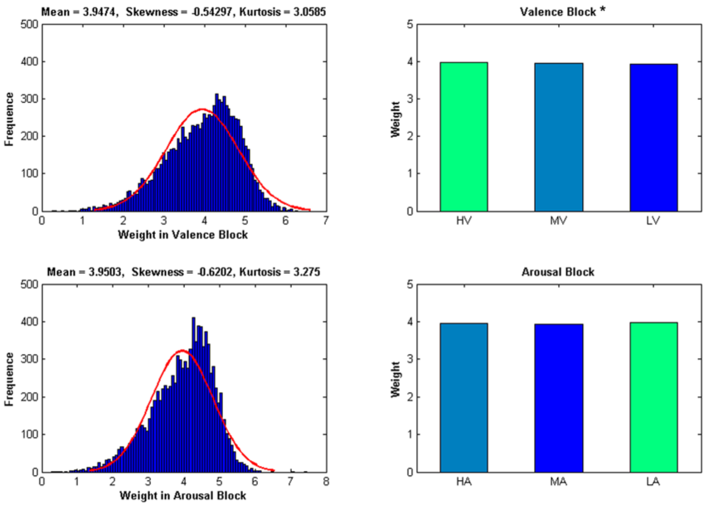

Figure S2. Edge weights statistics. Edge weights showed no marked difference between the valenced and aroused pictures (p=0.82, Welch's t-test). The weight of valence block displayed negatively skewed distribution, which was significantly different from the normal distribution ($p<0.001$, Jarque-Bera normality test). The weights in HV, MV, and LV showed marginal marked within-block difference (p=0.047, ANOVA). Trend analysis revealed significantly linear trend within three graded valence levels ($F(1, 3378) = 5.94$, p=0.015). On the other hand, the weight in the

arousal block also showed a negatively skewed distribution which was significantly different from the normal distribution (p<0.001, J-B test). The within block difference in HA, MA and LA was not significant (p=0.12, ANOVA), while the quadratic trend over three arousal levels was marginal significant (F(1,3080) = 3.28, p=0.07).

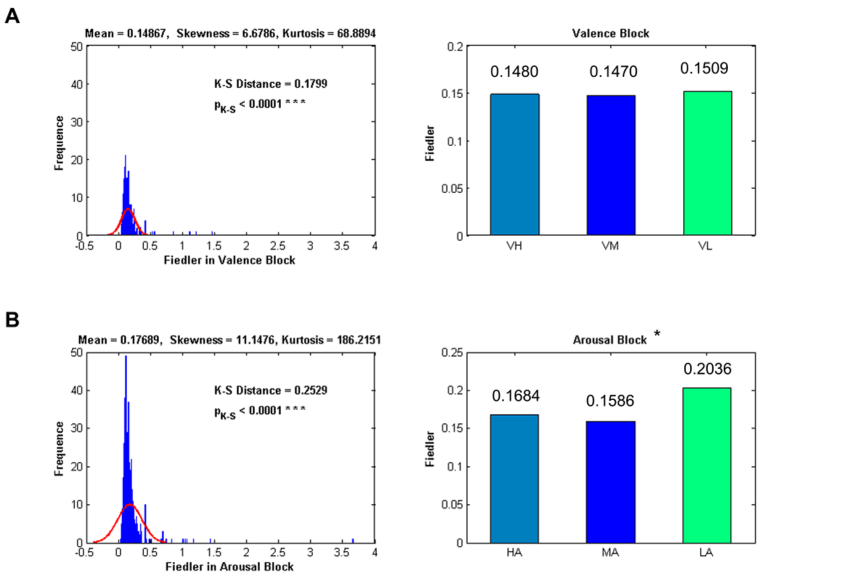

Figure S3. The Fiedler metric. The density distribution of Fiedler in valence and arousal blocks showed positively skewed leptokurtic patterns, which are significantly different from the normal distribution (both p<0.0001, Kolmogorov-Smirnov test). In addition, the Fiedler distribution was more positively skewed and more 'thin' in aroused pictures compared to valenced pictures. In the aroused pictures, the Fiedler demonstrated significant difference across three level (p<0.05, ANOVA), indicating a non-linear effect of the arousal dimension.

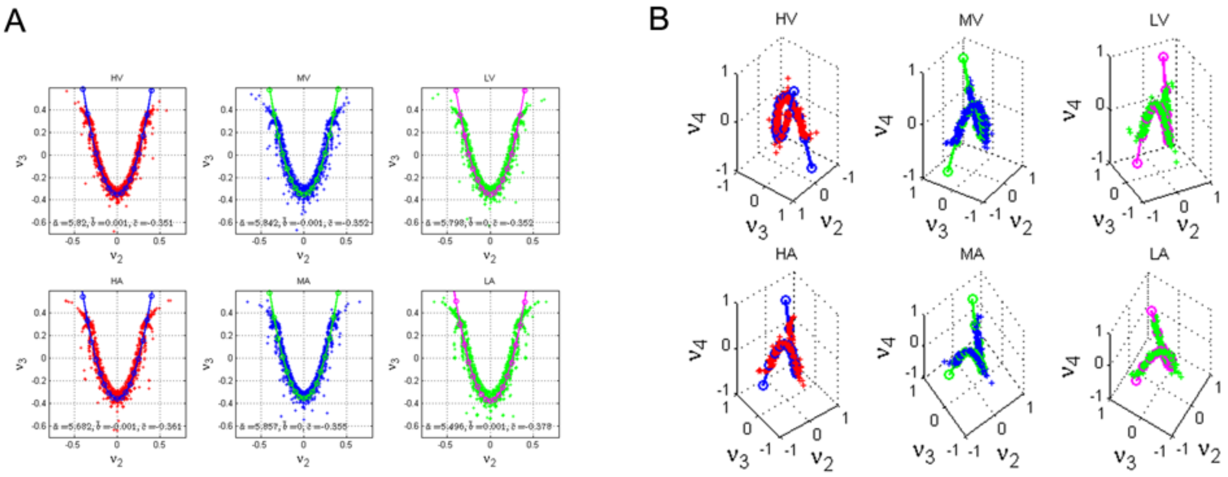

Figure S4. Spectral embedding of Scan-paths. (A) $\mathbb{R}^2$ spectral embedding displays a parabola-like curve, which could be fitted by quadratic function $\boldsymbol{v_3=\alpha\,(v_2-b)^2+c}$. (B) $\mathbb{R}^3$ spectral embedding of scan-path could be fitted by cubic curve function $\boldsymbol{v_4=\beta_0+\beta_1 v_2^{3}+\beta_2 v_3^{2}+\beta_3 v_2 v_3+\beta_4 v_2^{2}}$.

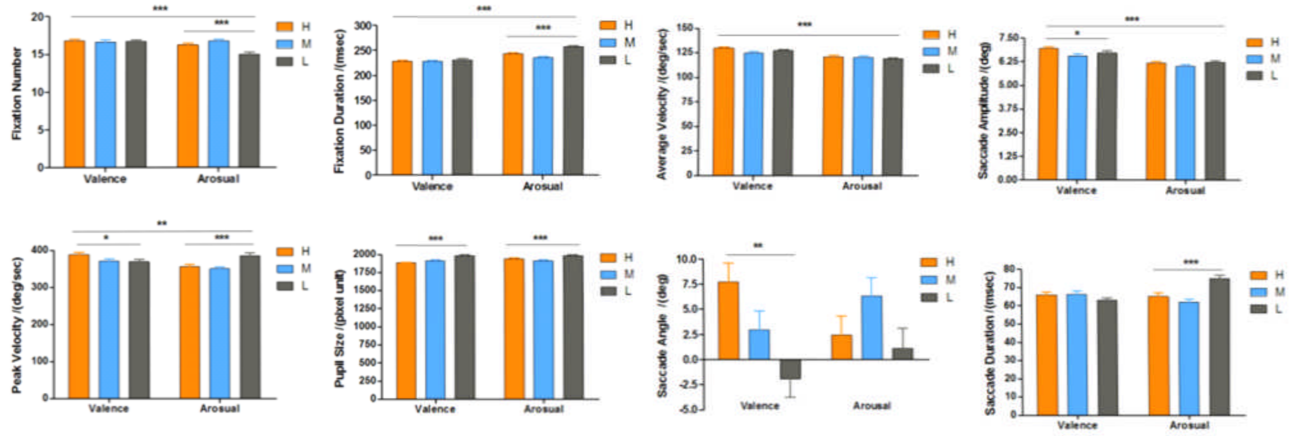

Figure S5. **Kinetic scan path metrics**. Fixation number (FNum), fixation duration (DRA), average velocity (AVG), saccade amplitude (SAP) and peak velocity (PKV) showed difference between aroused and valenced pictures (by Welch's t-test). In the arousal block, metrics such as FNum, DRA, PKV, PUL (pupil size) and saccade duration (SDR) indicated non-linear arousal effect; in the valence block, metrics such as PKV, PUL and saccade angle (SAG) indicated linear valence effect (by ANOVA). One star indicate $p<0.05$; two-star indicate $p<0.01$; and three-star indicate $p<0.001$;

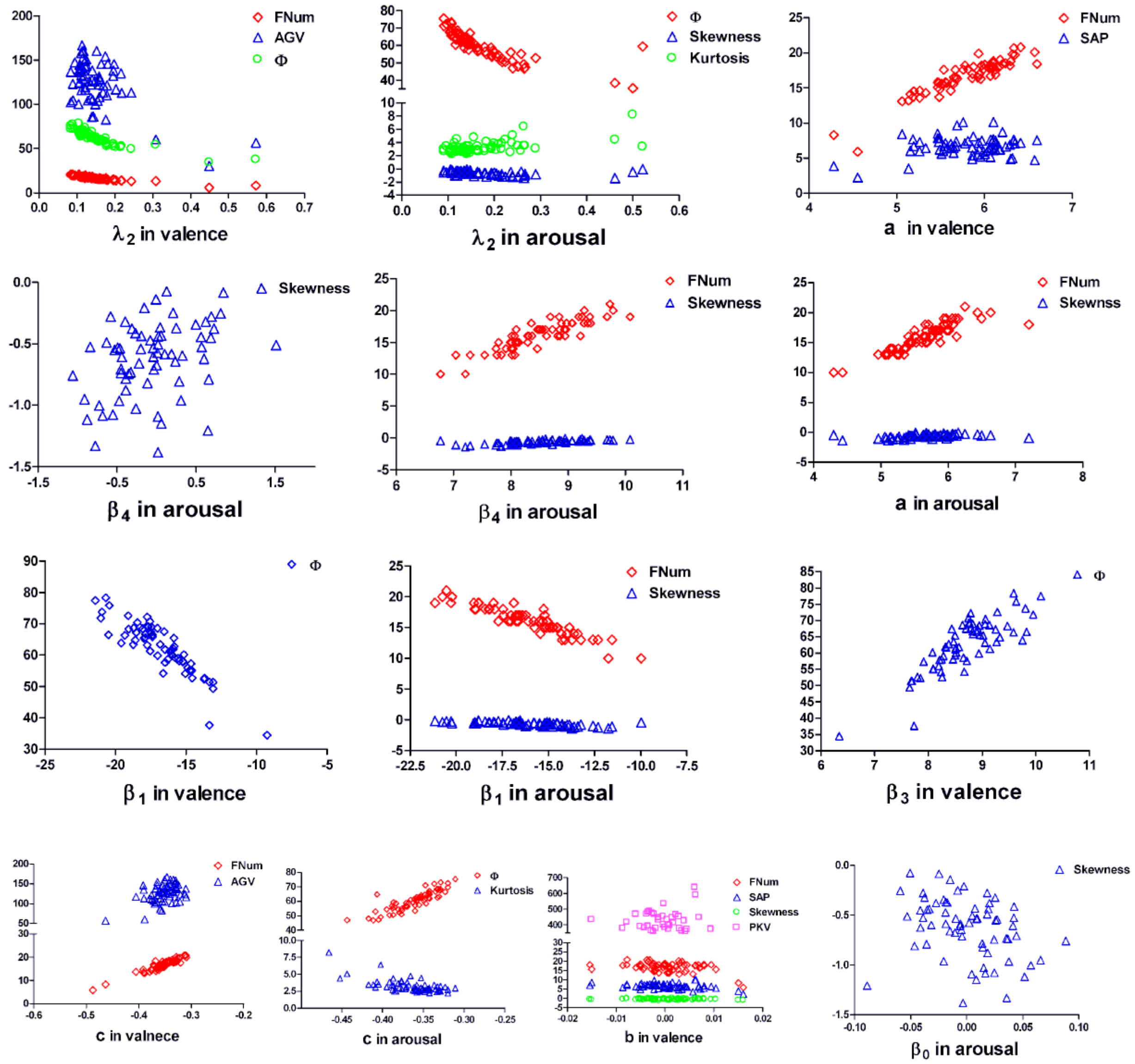

Figure S6. **Partial regression plot.** The dependent (N = 66) included metrics such as Fiedler, coefficients ***a, b, c*** , and coefficients β_0, β_1, β_3, β_4 in each affective level, respectively; the multivariate independents (10×66) are metrics including FNum, Φ, AGV, SDR, SAP, DRA, PKV,SAG, SKW (skewness of weight) and KUR (kurtosis of weight) in each affective level.

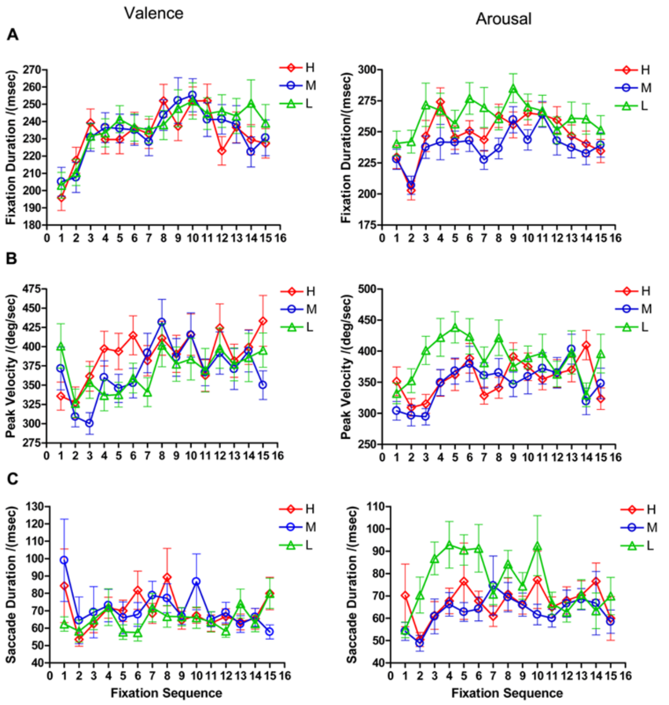

Figure S7 Time course of kinetic scan-path metrics. The trend of fixation duration (DRA), peak velocity (PKV) and saccade duration (SDR) plotted by the first 15 consecutive fixations in scan path, which pooled the data of 22 subjects. Curve trends were compared in three levels (H = High, M = Medium and L = Low). (A) Fixation duration. In the arousal block, the curve of high level is between medium level and low level, displays a non-linear effect in the arousal dimension ($F(1,14)=66.02$, $p<0.01$). (B) Peak velocity. In the valence block, the curve of high level is above the medium and low level one ($F(1,14)=5.74$, $p= 0.035$); in arousal block, the curve of low level is above the medium level and the low level ($F(1,14)=15.88$, $p=0.001$); (C) Saccade duration. In the arousal level, the high level curve is between the medium and the low level ($F(1,14)=15.295$, $p=0.002$). By Wilconxon matched pairs tests.
