# Supplementary material for: Dissociable Modulation of Overt Visual Attention in Valence and Arousal Revealed by Topology of Scan Path
Source: PLoS One. 2011 Apr 6;6(4):e18262. doi: 10.1371/journal.pone.0018262 (PMC3071806; doi:10.1371/journal.pone.0018262)
Supplement: Table S1 — The linear effect in valence. The linear valence effect was test by ANOVA and Turkey's post hoc test. H = high affective level; M = medium affective level; L = low affective level. TCC_PUL = pupillary reflex curves; TCC_PKV = time course curve of PKV. (PDF) [file pone.0018262.s002.pdf]

Table S1 The linear effect in valence

Metrics	Graded Valence Effect	Linear Trend Test
Weight	H > M > L	$F_{(1,3378)}$=5.944, p=0.015
SAP	H> M > L	$F_{(1,3450)}$=2.651, p=0.104
PKV	H > M > L	$F_{(1,3450)}$=6.237, p=0.013
SAG	H > M > L	$F_{(1,3450)}$=13.616, p=0.000
PUL	H < M < L	$F_{(1,3667)}$=88.190, p=0.000
TCC_PUL	H < M < L	$F_{(1,14)}$=66.022, p =0.000
TCC_PKV	H > L, H > M	$F_{(1,14)}$=5.471, p=0.035

The linear valence effect was test by ANOVA and Turkey's post hoc test. H = high affective level; M = medium affective level; L = low affective level. TCC_PUL = pupillary reflex curves; TCC_PKV = time course curve of PKV.
