# Supplementary material for: Dissociable Modulation of Overt Visual Attention in Valence and Arousal Revealed by Topology of Scan Path
Source: PLoS One. 2011 Apr 6;6(4):e18262. doi: 10.1371/journal.pone.0018262 (PMC3071806; doi:10.1371/journal.pone.0018262)
Supplement: Table S2 — The nonlinear effect in arousal. The nonlinear effect was test by ANOVA and Turkey's post hoc test. H = high affective level; M = medium affective level; L = low affective level. TCC_PUL = pupillary reflex curve; TCC_DRA = time course curve of DRA; TCC_PKV = time course curve of PKV; TCC_SDR = time course curve of SDR. (PDF) [file pone.0018262.s003.pdf]

Table S2 The nonlinear effect in arousal

Metrics	Graded Arousal Effect	Quadratic Trend Test
Weight	M < H < L	F(1,3378)=3.278, p=0.070
Diameter	M > H > L	F(1,214)=29.057, p<0.001
Fiedler	M < L	F(1,216)=2.291, p=0.132
ξ_2	M < L	F(1,216)=2.560, p=0.111
ξ_3	M < L	F(1,216)=3.688, p=0.056
a	M > L, H > L	F(1,216)=23.139, p<0.001
c	M > L, H > L	F(1,216)=13.348, p<0.001
β_1	M < L, H < L	F(1,216)=10.950, p=0.001
β_3	M > L	F(1,213)=3.924, p=0.049
SDR	M < H < L	F(1,213)=18.911, p<0.001
DRA	M < H < L	F(1,3308)=33.717, p<0.001
PUL	M < H < L	F(1,3308)=6.566, p=0.010
FNum	H > L, M > L	F(1,219)=32.010, p<0.001
PKV	M < H < L	F(1,3089)=8.907, p=0.003
TCC_PUL	M < H < L	F(1,14)=59.078, p<0.001
TCC_DRA	M < H < L	F(1,14)=51.830, p<0.001
TCC_PKV	M < L, H < L	F(1,14)=15.877, p=0.001
TCC_SDR	M < H < L	F(1,14)=15.295, p=0.002

The nonlinear effect was test by ANOVA and Turkey’s post hoc test. H = high affective level; M = medium affective level; L = low affective level. TCC_PUL = pupillary reflex curve; TCC_DRA = time course curve of DRA; TCC_PKV= time course curve of PKV; TCC_SDR = time course curve of SDR.
