# Supplementary material for: Dissociable Modulation of Overt Visual Attention in Valence and Arousal Revealed by Topology of Scan Path
Source: PLoS One. 2011 Apr 6;6(4):e18262. doi: 10.1371/journal.pone.0018262 (PMC3071806; doi:10.1371/journal.pone.0018262)
Supplement: Table S3 — IAPS Series Number of Pictures. (PDF) [file pone.0018262.s004.pdf]

Table S3 IAPS Series Number of Pictures

BLOCK		Female Subjects	Male Subjects
Valence	H	2040, 1710, 2347, 5833, 2550, 1811, 1463, 1590, 4574, 7430	1440,2150,2340,5825,8420, 1710,2154,2398,7200,8510
	M	8211,2351,8260,1560,2122, 1980,8065,2780,1645,8232	1101,1645,2458,3550.2,7248, 1390,2220,3210,6800,7497,
	L	9295,2799,9560,2900.1,9830, 2751,3181,2375.1,9435,2205	2095,3015,3064,3180,9185, 2800,3063,3101,9040,9405
Arousal	H	8179,8160,8178,1321,1931, 5950,8475,8341,8206,3250	1120,1931,8065,8160,8192, 1321,5940,8158,8191,8475
	M	7497,7211,4613,2372,7488, 7137,5455,1313,4631,7504	1240,2704,4613,7476,8041, 1303,4233,6940,7620,8232
	L	7059,2381,7187,7060,7491, 7020,7000,7010,7004,7175	2501,7004,7010,7080,7175, 5800,7006,7020,7110,7900

H = High level; M = Medium level; L = Low level.
